# AI solutions for adolescent psychiatry

**DOI:** 10.1192/j.eurpsy.2025.240

**Published:** 2025-08-26

**Authors:** T. Gargot

**Affiliations:** Child and Adolescent Psychiatry, University Hospital Tours, Tours, France

## Abstract

**Abstract:**

There is a potential for new technologies in mental health and psychiatry. Artificial intelligence enables the design models that categorize different groups and predict different prognosis trajectories.

Natural language processing enables to use classical text data from electronic health records, for instance, to detect suicide trends and their risk factors (Bey et al., 2024). This opens new perspectives in the analysis of large Electronic Health Records databases.

Artificial intelligence can extract and combine new features, like posture, physiological signal of stress and facial expression. This could be particularly important to bypass insight development in children of adolescents (Bourvis et al., 2021) while taking the opportunity of early management. This could help to optimize exposure therapy (Mahmoudi-Nejad et al., 2024), detect tantrums in non-verbal children (Cano et al., 2024) or even improve motivation for physical activity (Nuss et al., 2020).

In motion assessment, we could detect motor assessment difficulties in children with autism from typical counterparts (Gargot et al., 2022). We can also automatically detect writing difficulties (Agarwal et al, 2023).

However, AI struggles with an interpretability problem (black box). Their model are complex, the features extracted are not always obvious (Minh et al, 2021 ; Linardatos et al, 2020) .

Fine motor skills classic signal processing allows to tailor specific exercises to reeducate children with writing difficulties (Gargot et al., 2021).

Digital psychiatry however is impeded by poor user experience (Witteman et al., 2011), complex market models (Gollier-Briant et al., 2024).

**Disclosure of Interest:**

None Declared

